# Somatic Cough Syndrome in a Male Child: A Case Report

**DOI:** 10.7759/cureus.32767

**Published:** 2022-12-21

**Authors:** Moksha Prasoona, Faheem Vellekkat, Amreen Sait, Krishnan Gireesh, Vivek Sanker

**Affiliations:** 1 Internal Medicine, Gandhi Medical College, Secunderabad, IND; 2 Psychiatry, Indira Gandhi Medical College & Research Institute, Malappuram, IND; 3 Psychiatry, Mahatma Gandhi Missions (MGM) Medical College, Aurangabad, IND; 4 Clinical Psychology, Trivandrum Medical College Hospital, Trivandrum, IND; 5 General Surgery, NIMS Medicity, Trivandrum, IND

**Keywords:** mindfulness, cbt, cough, somatic cough syndrome, psychogenic cough

## Abstract

A 10-year-old boy, presented with a one-year history of persistent cough, insidious in onset, which often exacerbated to vomiting, perspiration, and breathlessness. Symptoms were exacerbated in school when a teacher was present in the classroom and relieved when he was occupied with hobbies such as watching television or just relaxing. He was prescribed multiple medications for over a year, but his symptoms persisted and did not show any improvement even after the use of appropriate medicines as per the doctor's advice. Despite detailed medical evaluation, no organic cause was found. So considering a non-organic cause, a psychological assessment was done and he was found to have an anxiety disorder (Child Anxiety Related Disorders questionnaire score of 26). He was initiated on cognitive behavioral therapy, mindfulness, and distress tolerance skill lessons. Subsequently, he showed significant improvement in his symptoms. This case emphasizes the necessity to recognize underlying anxiety disorder and psychosocial problems such as dysfunctional parenting in the management of persistent chronic cough with no identifiable organic cause.

## Introduction

Cough is one of the most common presenting complaints seen in patients of all ages with varying etiologies. Recently, the task force of the European Respiratory Society (ERS) suggested two definitions of cough: (i) A three-phase expulsive motor act is defined as having an inspiratory effort (inspiratory phase), a forced expiratory effort (compressive phase), the opening of the glottis, and a quick expiratory airflow (expulsive phase) [1]. The majority of the literature uses this concept, with the addition of a fourth "healing phase" (the profound inspiration that typically follows a cough) [1]; (ii) "A forced expiratory maneuver, typically with a closed glottis and accompanied by a distinctive sound” [1].

The type of cough can be identified by its cause, characteristics such as wet and dry, hacky, or chesty, duration or location of origin from the airways due to respiratory pathology or from the cerebral cortex [1]. One of the most curious cases in the medical field is that of psychogenic cough. The term "psychogenic cough" has been used to characterize coughs that lack a clear medical cause, are resistant to treatment, and are thought to have a psychiatric or psychological cause [2]. However, a change in guidelines by the American College of Chest Physicians (ACCP) in 2006 suggested the replacement of the term psychogenic cough with the term somatic cough syndrome to align with the terminology used in the Diagnostic Statistical Manual of Mental Disorders-5th edition (DSM-5) [2].

Diagnosis of somatic cough syndrome (psychogenic cough) is challenging, considering the lack of extensive studies, diagnostic tests, or criteria (inclusion or exclusion). Symptoms such as barking or honking cough with the absence of nocturnal cough were used to diagnose the condition until the 2006 ACCP guidelines, which recommended otherwise based on lack of specificity [2]. Similarly, the presence of anxiety or depression should not be used as a diagnostic criterion, as these psychological symptoms can arise because of persistent troublesome untreatable illness [2]. Some of the causes might have to do with semantics and/or a lack of distinction between habit coughing, tics, and Tourette syndrome. The majority of patients with Tourette syndrome and associated tic disorders are youngsters, with the average age of onset of tics being five to six years old. Tics are typically at their worst by age 10, and 50% of patients are rid of tics by the time they are 18 years old. A diagnosis of habit cough or psychogenic cough should not be established unless Tourette syndrome and other disorders linked to vocal tics have been specifically ruled out because the condition is frequently misinterpreted [3]. Here, we have a case of a 10-year-old boy with complaints of persistent cough for more than a year's duration.

## Case presentation

A 10-year-old male, who was a student in the fifth grade of his schooling, presented with more than a one-year duration of persistent cough along with acute pain in his right hand for one-month duration. His symptoms were absent before the onset of the pandemic and during the lockdown period. There was no history of domestic violence, child abuse/neglect, or any other significant medical history. There was no history of similar complaints or any other psychiatric conditions in his family. His family consists of five members: his father, mother, elder sister, and maternal grandmother. He tends to spend most of his time with his grandmother who, according to his parents, coddles and pays extra attention to the child.

Once the school recommenced, his symptoms started insidiously. The cough was persistent in nature, often resulting in vomiting, perspiration, and breathlessness. It was not associated with dizziness, loss of consciousness, fainting spells, palpitations, or visual disturbances. The child seemed anxious in the presence of a teacher. One perpetuating factor, which was elicited during the history-taking, showed that his symptoms were exacerbated whenever a teacher was present in the classroom. The episode of cough was often associated with pain in the right hand, which was acute, non-radiating, and constant dull aching in nature. He revealed that he was at utmost comfort with just his peers in the classroom as well as at home. Whenever he is occupied with hobbies such as watching television or just relaxing, his symptoms are very mild or absent. Past treatment history includes consultations with doctors from different specialties, including a general physician, a respiratory medicine/chest physician, and a neurologist. Despite being on multiple medications such as antihistamines, antibiotics, analgesics, and cough suppressants over the past year, his symptoms persisted and did not show any improvement. Multiple radiological investigations such as X-rays, Computed tomography (CT), ultrasonography (USG), and lab tests, such as complete blood count (CBC), routine sputum examination and culture, inflammatory markers, and patch test for allergy, were performed, yet none of them could yield a definitive diagnosis. Electrocardiography was done to rule out any cardiac causes, and it was found to be normal. This led to speculation of an absent organic cause.

A mental state examination revealed a boy with proper hygiene and grooming with decreased flow and volume of speech. His mood was euthymic and with anxious affect. No perception abnormality nor any disorders of thought could be elicited. His cognitive function was also intact. Application of the Screen for the Child Anxiety Related Disorders questionnaire yielded a total score of 26 out of 40, indicating the presence of anxiety disorder. Before beginning psychotherapy, he underwent a number of psychological tests, including The Child's Apperception Test, which demonstrated anxiety about being separated from attachment figures, and the Milan's Intelligence Scale for Indian Children, which revealed an IQ of 104 (Average). The therapeutic design was weekly sessions of CBT - cognitive behavioral therapy besides mindfulness and distress tolerance skill lessons. Subsequently, after five sessions of CBT, he showed significant improvement in his symptoms, which was observed both by himself and his parents. They noticed that he did not have a persistent cough and only complained of occasional pain in his right hand. His parents and the maternal grandmother were given counseling regarding how to approach the child during such episodes. He feels a lot better symptomatically and is now under follow-up.

## Discussion

Youngsters, teenagers, and, uncommonly, adults have been reported to experience habitual coughs or "psychogenic" coughs. Usually, an upper respiratory tract infection causes a cough, but that cough lasts for a long time after the other respiratory symptoms go away. Rarely do people with persistent cough develop a psychogenic cough [4]. Usually chronic, it interferes with daily life and results in long-term distress [5]. There is no clinical or laboratory indication of disease, in contrast to the cough that is associated with organic etiologies [6]. The term "psychogenic cough" typically refers to a somatization illness unrelated to conversion disorder and malingering. Somatization is the conversion of a psychological symptom into a physical embodiment [2]. Only after a thorough assessment, which includes ruling out tic disorders, could psychogenic cough be diagnosed [7]. The absence of a physical reason distinguishes habit coughs from other types of coughs. The majority of the time, psychogenic coughs appear after a child has been coughing for another cause (such as a cold or chest infection). It may become habitual for the individual to continue doing so, even when there is no need to cough.

The ACCP standards indicated that a diagnosis of somatic cough syndrome (psychogenic cough) can only be determined if the patient qualifies the DSM-5 criteria for a somatic symptom disorder, a thorough evaluation has been completed, and the common causes of cough have been ruled out. One or more somatic symptoms that are distressing or significantly interrupting everyday life are included in the DSM-5 criteria. There could be exaggerated and persistent worries about how serious the symptoms are, a lot of anxiety over them, or excessive time and focus spent on them [8]. Theorists of psychodynamic and behavioral psychology have made attempts to pinpoint the root cause of the somatic cough syndrome's vicious cycle. While Linden Baum and Clark observed that the cough fits were a means for some children to communicate repressed anger and disappointment, Bernstein defined the cough as a "bark-out" protest against a domineering mother [9]. Diagnostically, this syndrome presents as a chronic, loud cough with a barking or honking quality that substantially disrupts patients' lives but fades while they hit the sack [10]. With escitalopram, a selective serotonin reuptake inhibitor (SSRI) exhibiting a favorable side effect profile in long-term therapy, studies have emphasized the relevance of SSRIs in the management of concomitant anxiety disorders in these situations [11].

There is no concrete evidence to support a specific method for defining, diagnosing, and treating psychogenic, habitual, and tic cough [7]. For the treatment of somatic cough syndrome, both pharmacological and nonpharmacological modalities were tested; the latter was thought to be more effective, albeit there are still no randomized head-to-head comparisons between modalities [2,7]. The most advised forms of intervention in these circumstances, according to a systematic review by Haydour et al. comprised suggestion therapy, hypnosis, reassurance, and counseling. It showed seven studies that combined several types of therapies, such as counseling, relaxation exercises, referral to a psychologist, and psychotherapy, and 93% of individuals who underwent these therapies saw an improvement in their cough [7]. It is also reported that there was generally little benefit from pharmacological therapies [7,10,11]. Additionally, no medications have been approved to treat somatic cough syndrome, and those that have been are usually only given to those who also have other psychiatric comorbidities. A case series of four children with somatic cough syndrome by Shahzadi and Shweta showed a significant improvement in their symptoms with CBT [12]. The present study also addresses the method of controlling psychogenic cough in children using CBT.

It is also important to identify comorbid anxiety disorder, depression, and psychosocial issues, such as punitive parenting, in addition to the persistent chronic cough and the observation that these associated comorbidities were successfully managed while the cough was controlled suggests that, in at least some of these instances, the cough may only be a symptom of the comorbidity rather than a separate diagnosis [2,11,13]. Chronic cough and psycho-morbidity have a tangled link. The description of "psychogenic cough" implies that psychosocial concerns have a role in the etiology of persistent cough. Alternatively, in certain people, a persistent cough might lead to psycho-morbidity. In primary care, the therapy for persistent cough fluctuates, and patients may go through protracted, fruitless tests and therapeutic trials in an attempt to pinpoint and treat the symptoms [14].

## Conclusions

Diagnosis of psychogenic cough most likely is one of exclusion. Before diagnosing a pediatric patient with psychogenic cough, it is necessary to rule out tic disorders including Tourette syndrome. It is important to evaluate for coexisting conditions, such as anxiety (as in this case), depression, domestic violence, and child abuse/neglect, and manage them accordingly. Diagnosing and treating psychogenic cough early can significantly improve the outcome and prevent needless interventions and iatrogenic complications. Patients with psychogenic cough show significant improvement with psychotherapy or behavior modification.
